# A Robust Personalized Classification Method for Breast Cancer Metastasis Prediction

**DOI:** 10.3390/cancers14215327

**Published:** 2022-10-29

**Authors:** Nahim Adnan, Tanzira Najnin, Jianhua Ruan

**Affiliations:** Department of Computer Science, The University of Texas at San Antonio, 1 UTSA Circle, San Antonio, TX 78249, USA

**Keywords:** cancer metastasis, personalized classifier, gene expression, biomarker discovery

## Abstract

**Simple Summary:**

Accurate prediction of breast cancer metastasis risks using gene expression data and machine learning can help improve cancer treatment and overall survival. However, breast cancer can be categorized into multiple subtypes, and a single predictive model may not work well for all patients. In this work, we propose a computational method to construct personalized models, where the key is to select a group of patients to train a different model for each testing patient. Experimental results on multiple datasets showed that the proposed method, termed Personalized Classifier with Multiple Thresholds (PCMT), achieved significantly better prediction accuracy than existing algorithms that train classifiers using all available patients or using patients belonging to a predefined subtype. In addition, the top features identified by PCMT are robust across different datasets, and include genes that are well known to be associated with subtype-specific metastasis.

**Abstract:**

Accurate prediction of breast cancer metastasis in the early stages of cancer diagnosis is crucial to reduce cancer-related deaths. With the availability of gene expression datasets, many machine-learning models have been proposed to predict breast cancer metastasis using thousands of genes simultaneously. However, the prediction accuracy of the models using gene expression often suffers from the diverse molecular characteristics across different datasets. Additionally, breast cancer is known to have many subtypes, which hinders the performance of the models aimed at all subtypes. To overcome the heterogeneous nature of breast cancer, we propose a method to obtain personalized classifiers that are trained on subsets of patients selected using the similarities between training and testing patients. Results on multiple independent datasets showed that our proposed approach significantly improved prediction accuracy compared to the models trained on the complete training dataset and models trained on specific cancer subtypes. Our results also showed that personalized classifiers trained on positively and negatively correlated patients outperformed classifiers trained only on positively correlated patients, highlighting the importance of selecting proper patient subsets for constructing personalized classifiers. Additionally, our proposed approach obtained more robust features than the other models and identified different features for different patients, making it a promising tool for designing personalized medicine for cancer patients.

## 1. Introduction

Breast cancer is a leading cause of cancer-related death in women, while the majority of breast cancer patients have a good prognosis after therapy, about 30% of early-stage breast cancer patients will experience distant metastases, and 90% of patient deaths are because of complications from metastases [1]. High-throughput gene expression profiling by microarray or next-generation sequencing has played a significant role in identifying biomarkers to predict metastasis [2,3,4]. Additionally, integration of gene expression data with network information such as protein–protein interaction network, metabolic network, gene-gene co-expression network, and molecular pathways have resulted in more accurate prediction models, stable and biologically interpretable features, or both [5,6,7,8,9,10,11,12,13,14,15]. The accuracy of these prediction models is often comparable to or better than conventional histological grading-based methods [16,17,18].

It is well established that cancer is a heterogeneous disease, which hinders the overall performance of metastasis prediction models. Breast cancer can be commonly divided into five subtypes (i.e., basal, HER2, luminal-A, luminal-B, and normal-like), each categorized by different molecular characteristics and prognosis [17,19,20,21,22,23]. However, the subtype definition can vary, depending on the gene signatures used. Importantly, the molecular profile for patients within the same subtype can still show significant diversity. To address the heterogeneity issue, Jahid et al. proposed an ensemble-based method, a personalized committee classifier, for metastasis prediction [24], which achieved better performance than other ensemble approaches. The basic idea is to generate a vast pool of classifiers using different groups of patients as training data and select an appropriate subset of classifiers from the pool for each patient to be predicted. However, the algorithm depends on several critical parameters for model selection, which is hard to tune and does not scale well to larger datasets because of the large number of classifiers to be generated. In addition, due to its complexity, the prediction model for each patient is hard to interpret.

In this paper, we propose a novel method, termed personalized classifier with multiple thresholds (PCMT), to address the issues mentioned above. To predict the metastasis risk for a target patient, the algorithm first selects a group of training patients whose similarities to the target patient are the highest or lowest, and then train a classifier using only these selected patients to make a prediction for the target patient. Our results showed that PCMT achieved significantly better prediction accuracy compared to logistic regression and random forest models trained on the whole dataset in several cross-validation schemes using multiple independent datasets. Additionally, PCMT outperformed subtype-specific models trained only on patients of the same subtype. Our analysis also revealed that including the most similar and the least similar patients in training the personalized classifier improved the prediction accuracy of PCMT compared to classifiers trained only on the most similar patients. Finally, we showed that personalized classifiers improved robustness in identifying top features compared to the base models and that the personalized classifier was able to generate different top genes for different subtypes, which was not possible by using the base models.

## 2. Methods

### 2.1. ACES Dataset

For model training and testing, the gene expression dataset is collected from the Amsterdam Classification Evaluation Suite (ACES) [25]. This dataset is compiled from twelve separate breast cancer cohorts from NCBI’s Gene Expression omnibus. Only the Affymetrix HG-U133a microarray platform was used in this compiled dataset, and duplicated samples with the same GEO id were excluded from multiple cohorts. Details and preprocessing steps are given in [25]. Briefly, sample array quality control was checked for all the patients from the same study using R’s arrayQualityMetrics; outlier samples were excluded from further consideration using RLE (Relative Log Expression) or NUSE (Normalized Unscaled Standard Error Plot) analysis; data is then normalized by justRMA method from R, followed by log normalization and mean centered normalization. Finally, R’s combat method was used to remove batch effects from the final cohort. The final dataset contains the expression levels of 12,750 genes for 1616 patients. A patient is recognized as having a good/negative/non-metastatic outcome if the patient is free from relapse within five years; otherwise, the patient is recognized as having a poor/positive/metastatic outcome. In the overall ACES dataset, there were 455 metastatic outcomes. Details for each cohort are given in Table 1.

### 2.2. NKI Dataset

To further evaluate the feasibility of our proposed algorithm, we used another dataset independent from the ACES dataset. The NKI gene expression dataset was obtained using the Affymetrix HG-U133a platform and contains 11,658 genes and 295 patients [2]. There are 10,398 common genes between the ACES and NKI datasets. The expression data of these common genes from the two datasets are quantile normalized together so that data from the two sources have the same distribution. Among the 295 patients in the NKI dataset, 78 developed metastasis within five years of initial diagnosis and are labelled as having poor outcomes, while the others are labelled as having good outcomes.

### 2.3. TCGA Dataset

To assess whether the classifiers built using microarray data can be used to make predictions on newer data generated with the RNAseq technology, we downloaded TCGA prognostic dataset [26]. We considered only primary solid tumors (sample_type_id = 01). The prognostic dataset includes the PFI.time attribute, which indicates the progression-free interval of the patient, alongside a boolean event status attribute, which indicates if an actual progression-related event has occurred and the patient simply stopped following up with the care providers. We define our prognostic outcome label as a function of these two attributes as follows:

$$\mathrm{outcome}\left(\mathrm{PFI}.\mathrm{time},\mathrm{event}\right)=\left\{\begin{array}{cc} \mathrm{good}, & \mathrm{if}\;\mathrm{PFI}.\mathrm{time}>5\;\mathrm{years}\\ \mathrm{poor}, & \mathrm{if}\;\mathrm{PFI}.\mathrm{time}\leq 5\;\mathrm{years}\;\mathrm{and}\;\mathrm{event}=1\\ \mathrm{undecided}, & \mathrm{otherwise} \end{array}\right.$$

For this study, we only considered patients with either “good” or “poor” outcomes. After applying these criteria, the final dataset had 225 patients with good outcomes and 123 with poor outcomes.

The TCGA gene expression dataset consisted of a total of 13,494 protein-coding genes, of which 9874 are common in the ACES dataset. The TCGA log2(norm + 1) data was first mean centered, and quantile normalization was applied to the combined TCGA and ACES dataset containing only the common genes.

### 2.4. Personalized Classifier with Multiple Thresholds (PCMT)

Cancer is a heterogeneous disease, meaning many different subtypes exist within the dataset. However, the patients with the same subtype are similar in molecular characteristics. Therefore, we define Personalized Classifier (PC) for a sample as a classifier built on a subset of the training samples selected according to their similarity with that specific sample. Patients with the highest similarity tend to have the same label, which may reduce the generalizability of the learned model. In addition, these patients may have distinct distributions in some feature values, and the resulting model may be sensitive to outliers in the test patient. We included the most similar and most dissimilar patients from the training data to solve this problem. Similarity is measured using the Pearson Correlation Coefficient (PCC) between two feature vectors, where each feature vector contains the expression levels of 12,750 genes for one patient. Other similarity measures may be used, depending on the dataset’s characteristics. To build a PC for a patient, we first calculate the PCCs for the entire training dataset. Then we filtered the similar and dissimilar patients based on a PCC threshold, estimated from the average number of samples in each training data subset resulting from the threshold. Next, the positively and negatively correlated patients whose absolute PCCs are greater than the PCC threshold are selected. Then a Logistic Regression (LR) model has trained on the filtered training patients using the expression levels of the 12,750 genes as independent variables and metastasis status as dependent variables. Finally, the testing patient is classified by the trained LR model. Details are given in Algorithm 1.

To build Personalized Classifier with Multiple Thresholds (PCMT), a set of PCC thresholds are used to cover a wide range of training data sizes, reflecting the uncertainty in subtype population size for each testing patient. The PCC thresholds in this paper have been chosen such that for different thresholds, sufficiently different training datasets can be selected. The complete ACES dataset has ∼1600 patients, which can be further grouped into five subtypes with sizes ranging from ∼100 to ∼600. Therefore, we chose six PCC thresholds from 0.15 to 0.275 with a 0.025 increase, which resulted in median training dataset sizes from 71 (PCC = 0.275) to 504 (PCC = 0.15). A smaller (less stringent) threshold would increase the training dataset size and may result in too many training patients and defeat the purpose of personalized classifiers. In contrast, a larger (more stringent) threshold would reduce the training dataset size and cause overfitting.

After the classifiers from different PCC thresholds are generated, the testing patient is classified by each of the trained PC classifiers, which generate predicted probabilities. The predicted probabilities from the multiple PC classifiers are averaged to obtain the predicted probability of PCMT for the testing patient. The algorithm details are given in Algorithm 2.
**Algorithm 1** Personalized Classifier.**Require:**X_train,X_test,pcc_threshold PCCs are calculated for each patient in X_train for X_test Identify the patients in X_train whose absolute PCC is greater than pcc_threshold Trained a logistic regression (LR) model using the identified patients **Return** the trained LR model

**Algorithm 2** Personalized Classifier with Multiple Thresholds (PCMT).
**Require:**X_train, X_test, a set of pcc_thresholds Get Personalized Classifiers for each of the pcc_threshold in the pcc_thresholds for X_train and X_test (i.e., Personalized Classifier(X_train,X_test,pcc_threshold)) Obtain predicted probabilities from each of the PC classifiers for X_test Average the predicted probabilities of the PC classifiers **Return** predicted probability for the X_test


### 2.5. Prediction Performance Comparison

To compare the prediction accuracy improvement of PCMT with the base classifiers, two models (i.e., Logistic Regression (LR) and Random Forest (RF)) implemented in the python scikit-learn package were considered. Default parameters were used in all experiments, except that for LR, the L1 penalty was set to true to achieve sparse solutions due to a large number of gene features present in the dataset. Previous studies in the same dataset and similar datasets have shown that RF has consistently outperformed other classifiers in predicting breast cancer metastasis, and its performance is robust concerning parameter variations, making it a preferred choice for benchmarking [24,27]. The area under the receiver-operating characteristics curve (AUC) score was used to determine the prediction accuracy of the prediction models. Performance measure using area under the precision-recall curve (AUPRC) had similar results and was not shown in the paper for clarity. Three types of cross-validation (CV) schemes were performed. First, 5-fold cross-validation (5-FCV) was repeated eight times, providing 40 AUC scores. Second, leave-one-study-out cross-validation (LOSO-CV) was performed, where one study was kept as a test set, and the other 11 studies were kept as the training dataset. LOSO-CV is used here to evaluate the algorithm’s performance in addressing the heterogeneity between different patient cohorts. Additionally, leave-one-out cross-validation (LOOCV) was performed to capture the prediction accuracy over the whole dataset. Finally, paired *t*-test was performed in 5-FCV and LOSO-CV, comparing PCMT with the individual PC classifiers and the base LR and base RF models.

To further evaluate the performance of PCMT and other classifiers, we used the ACES dataset as the training data, and the TCGA or NKI dataset as the testing data, while the experiment is similar to the LOSO-CV evaluation, here only a minimum cross-dataset normalization was performed between the training and testing data. In addition, the TCGA dataset was obtained using the RNAseq technology, while the ACES dataset was obtained using the microarray technology. Therefore, this experiment is expected to be more challenging than the LOSO-CV evaluation on ACES dataset, and may represent a more realistic clinical setting. We note that cross-dataset compatibility is itself a difficult challenge and therefore additional effort will be needed to further improve the cross-dataset normalization procedures in future studies.

### 2.6. Prediction Accuracy of Subtype-Specific Models

Another experiment we did was to measure the prediction accuracy for each pre-defined subtype of breast cancer. Patient in the ACES dataset are classified into five subtypes, i.e., basal, HER2, luminal-A, luminal-B, and normal-like, based on the expression patterns of pre-defined gene markers [25]. To evaluate subtype-specific classifiers, leave-one-out cross-validation (LOOCV) was performed with patients from each subtype using base LR and base RF models. In this way, each model had 5 AUC scores for the five pre-defined subtypes. On the other hand, to capture the AUC score of PCMT, leave-one-out cross-validation (LOOCV) was performed on the ACES dataset to generate the predicted probabilities for each patient belonging to the ACES dataset. Then, predicted probabilities for a subtype were taken into account, and the AUC score was calculated only using those predicted probabilities for that specific subtype.

### 2.7. Prediction Accuracy Improvement Using Negatively Correlated Patients

We performed an additional experiment to show the impact of including the negatively correlated patients in the PC classifier. As the baseline experiment, the results obtained from Section 2.5 were used, where both the positively and negatively correlated patients passing the PCC thresholds were included in the PC classifiers. An additional experiment was done for PCMT using only the positively correlated patients. Paired *t*-test was performed in 5-FCV and LOSO-CV to compare the PC classifiers trained on positively and negatively correlated patients with the PC classifiers trained on only the positively correlated patients for different thresholds of PC classifiers and PCMT.

### 2.8. Robustness Analysis of PC Classifier

The whole ACES dataset was randomly partitioned into two disjoint sub-samples. Then top *x* features were taken from both the sub-samples for robustness analysis. Finally, the robustness measure was defined as the ratio between the observed number of common top features from the two sub-samples and the expected number of common features by chance [28]. Since the expected number of overlaps between two lists of features is calculated as

$$\mathrm{expected}\_\#\_\mathrm{of}\_\mathrm{overlaps}=\frac{{\left(\#\_\mathrm{of}\_\mathrm{selected}\_\mathrm{top}\_\mathrm{features}\right)}^{2}}{\left(\mathrm{total}\_\#\_\mathrm{of}\_\mathrm{features}\right)}$$

The number of selected top features is determined by [28]:

(1) $$X=\sqrt{\mathrm{total}\_\#\_\mathrm{of}\_\mathrm{features}\times \mathrm{expected}\_\#\_\mathrm{of}\_\mathrm{overlaps}}$$

Splitting ACES into two sub-samples was repeated 20 times so that a vector of 20 overlap ratios was obtained for each model for a specific value of the expected number of overlaps. The number of expected overlaps was kept as 5, 10, 20, 30, and 50, so the top features chosen were 252, 357, 504, 618, and 798 from each model.

A single personalized classifier (PC) with a PCC threshold of 0.175 was used for the robustness evaluation. For a single partition of sub-samples, leave-one-out cross-validation (LOOCV) was performed for this specific PCC threshold. Then the learned coefficients were generated for each sample belonging to that partition. The average of the absolute coefficients was calculated to obtain a feature importance score from PC classifiers. The exact process was repeated for the other disjoint partition of sub-samples. This way, features with the highest average coefficient were selected from two disjoint sub-samples. The robustness measure was calculated by taking the ratio between the number of common top features and the number of expected overlaps. As a random control, another version of the personalized classifier was created using random neighbors while keeping the same number of patients as the actual personalized classifier with a PCC threshold set to 0.175. For base LR and base RF, two different models were trained on two disjoint sub-samples, and top features were selected from the model’s feature importance score. Random Forest inherently provides feature importance scores for the features. For LR, the absolute values of the learned coefficients were used for selecting the top features.

### 2.9. Top Gene Analysis from Personalized Classifiers

Model coefficients were generated from personalized classifiers using LOOCV, in which the threshold was set to 0.2. The coefficients of the patients belonging to a subtype are taken into consideration. Then feature ranking was generated by averaging the absolute coefficient. This way, 20 top genes were selected for five subtypes from a personalized classifier. Similarly, coefficients were generated by base LR using LOOCV. Finally, feature ranking was generated by taking the average of absolute coefficient for base LR. Top 20 genes were selected from base LR.

## 3. Results and Discussion

### 3.1. Prediction Accuracy of Personalized Classifier with Multiple Thresholds (PCMT)

**Figure 1:**
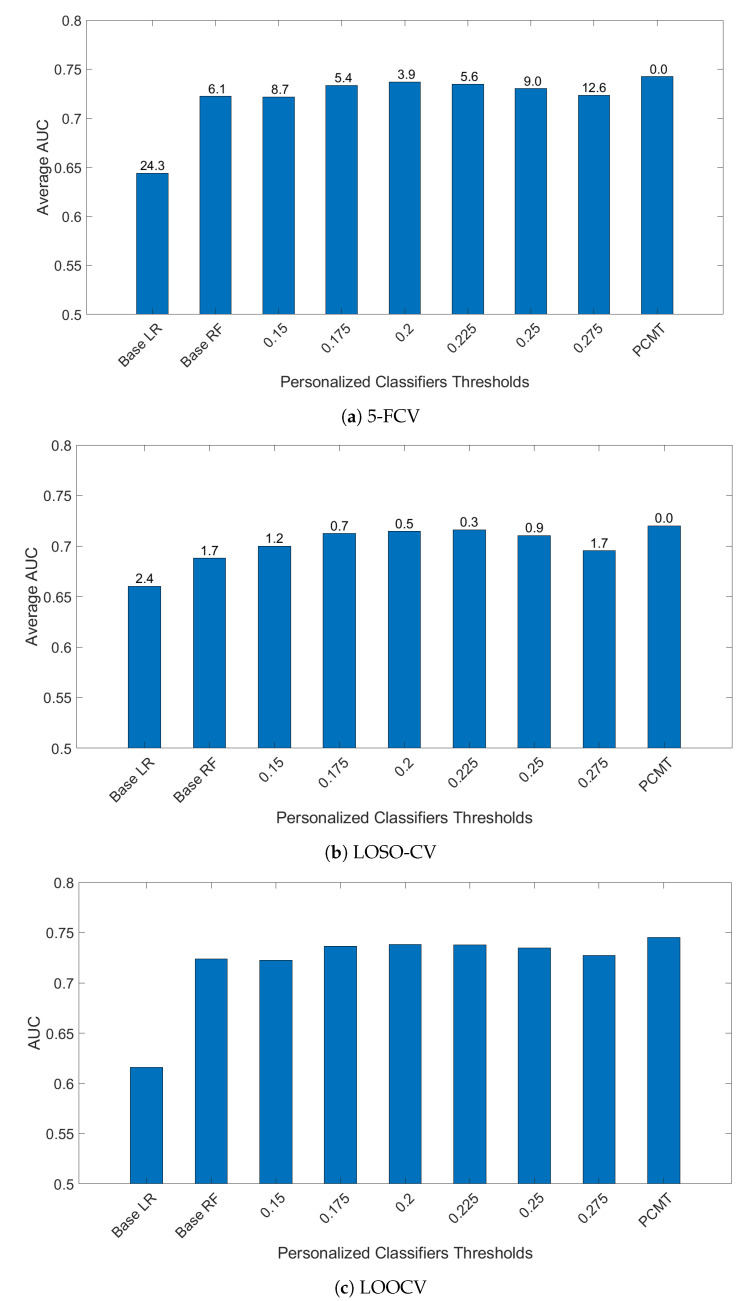
AUC comparison of personalized classifiers for different thresholds in (**a**) 5-FCV, (**b**) LOSO-CV and (**c**) LOOCV. Bar denotes the average AUC. Value on top of the bar denotes the −log10(p_value) of the paired *t*-test between PCMT and that corresponding bar.

To determine the effectiveness of PCMT in predicting metastasis, three different cross-validation schemes were employed. In 5-FCV settings, PCMT outperformed base LR and base RF as well as each of the single personalized classifiers considerably, shown in Figure 1. The mean AUC differences are statistically significant (paired *t*-test). In leave-one-study-out cross-validation (LOSO-CV), PCMT outperformed the base LR and RF models, and the differences are statistically significant. PCMT also achieved better performance than each PCs with limited statistical significance (Figure 1). In general, cross-dataset validation is a hard task due to dataset-specific characteristics that cannot be easily removed even with cross-dataset normalization. In addition, these datasets differ significantly in terms of dataset size and class distribution (Table 1), as well as subtype distribution (data not shown). When a relatively larger dataset is held out, there may not be enough number of similar patients in the training data to build an accurate predictive model. The results from LOOCV are very similar to that from 5 to FCV. In addition, we also measured the performance using Area Under Precision-Recall Curve in the LOOCV experiments, and the trend is almost identical to the results measured by Area Under ROC Curve (data not shown).

While PCMT and all the single PCs used LR as its base classifier, building a single classifier for all patients using LR performed much worse in all three validation schemes. This indicates that a single model for the whole dataset cannot capture the heterogeneity within the dataset.

**Figure 2:**
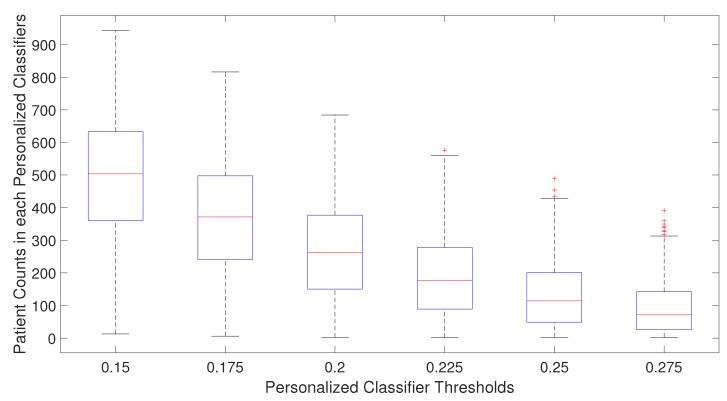
Number of patients used in training personalized classifiers for each patient for specific personalized classifier threshold.

For single personalized classifiers, the prediction accuracy is the highest for PCC threshold = 0.2 and much lower for PCC = 0.15 and PCC = 0.275, suggesting that a balance between training dataset size and heterogeneity in training data is needed for accurate prediction models. From Figure 2, it can be seen that the selected number of patients decreases as the PCC threshold increases. The median number of patients is around 70 for the PCC threshold of 0.275, which may be too small to build a good prediction model without overfitting. On the other hand, if the PCC threshold is too small, the number of training patients in each classifier may be too large, and the system will become similar to a single classifier for all patients. It will result in lower prediction performance as the heterogeneity of patient subtype characteristics is lost.

**Figure 3:**
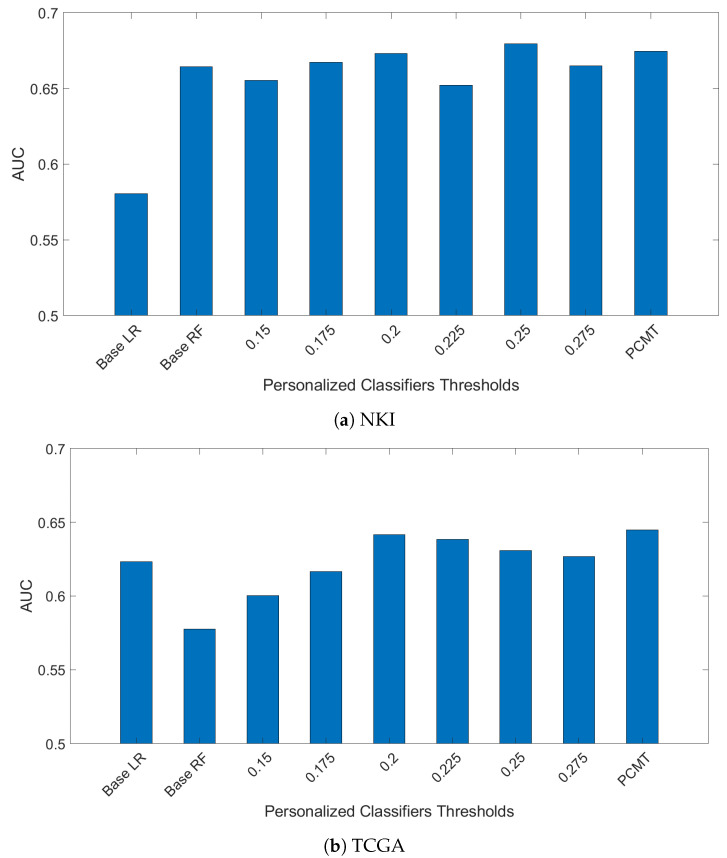
Models are train on ACES dataste only. Then mdoels were evaluted on two independent dataset TCGA and NKI breast cancer dataset.

In another experiment, models trained on the ACES dataset were evaluated using the TCGA and NKI datasets. The results are shown in Figure 3. The NKI dataset was produced with the same microarray platform as the ACES dataset, but the pre-processing steps are slightly different. The overall performance on the NKI dataset is similar to the LOSO-CV results on ACES. However, the relative performance of the single personalized classifiers with different thresholds shows a somewhat different trend than in the ACES dataset, suggesting a different patient composition in the NKI dataset. Regardless, PCMT achieved near optimal results without the burden of choosing the best threshold value.

On the TCGA data, PCMT achieved much higher prediction performance compared to the base LR, base RF and single personalized classifiers. On the other hand, the overall performance is much lower in all models compared to the performance on the ACES dataset. This is not surprising given that the TCGA data was generated with RNAseq technology, and improving the compatibility between microarray data and RNAseq data is itself a daunting challenge [29]. Nevertheless, the performance gain of PCMT mainly comes from its advantage in selecting relevant patients to train classifiers that are appropriate for the test patient’s molecular characteristics, which is relatively less affected by cross-dataset differences. Additional effort will be needed in future studies to improve the prediction performance in cross-dataset and cross-platform testing in a realistic clinical setting.

### 3.2. Running Time Comparison

One potential disadvantage of PCMT is that it needs to calculate the Pearson correlation coefficient between each testing sample and all the training samples, and train multiple classifiers for each testing sample. To evaluate its efficiency, we took note of the running time while performing leave-one-out cross-validation (LOOCV) for LR, RF, and PCMT. The total running time to complete the LOOCV is 17,697 s for LR, 8546 s for RF, and 6353 s for PCMT. As there are 1616 patients, this translates to about 11 s for LR and 5.3 s for RF to build a single model. The time taken to make predictions with LR and RF models is negligible. For PCMT, there are no pre-trained models. The average running time is 4 s per testing instance, which is spent on identifying training samples and constructing multiple LR classifiers with smaller training datasets than in LR or RF. In practice, as testing samples usually do not come in large batches, the increased running time in testing for PCMT should not be a major concern.

### 3.3. Prediction Accuracy Improvement by Including Negatively Correlated Patients in the Personalized Classifier

**Figure 4:**
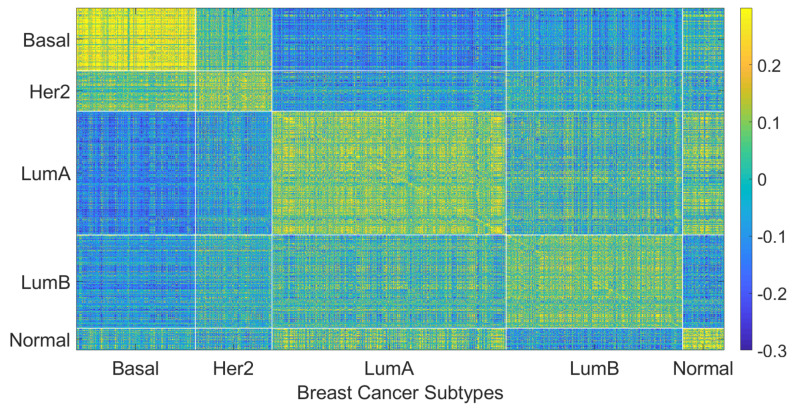
Patient to patient Pearson correlation coefficient of gene expression organized by breast cancer intrinsic subtypes.

**Figure 5:**
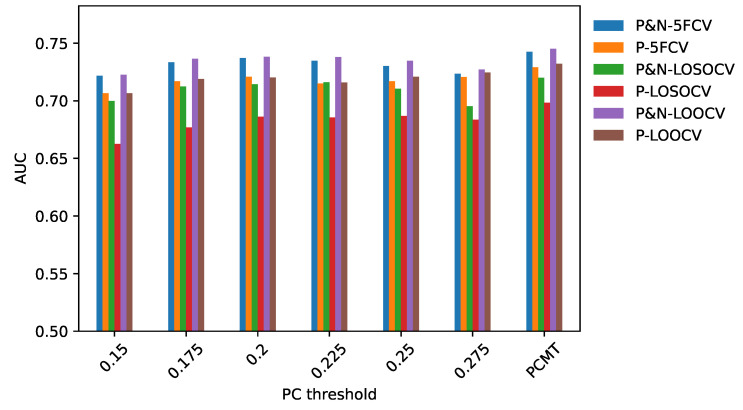
AUC comparison between personalized classifiers trained on positively and negatively correlated samples and personalized classifiers trained on only positively correlated samples.

We hypothesize that including negatively correlated patients in the training data increases diversity within the training data, which enables the personalized classifier to classify the testing patient better. Figure 4 shows correlation coefficients between patients within the same or different breast cancer intrinsic subtypes. It can be seen that the patients have a positive correlation among the patients of a specific subtype but have a low or negative correlation with the patients from different subtypes, which leads to the idea of including the negatively correlated patients in the personalized classifier. To show the effectiveness of including negatively correlated patients in the personalized classifiers, prediction accuracy was measured using the personalized classifiers built with only the positively correlated patients and including both positive and negatively correlated patients. The results are given in Figure 5. It can be observed that the personalized classifiers, both the single PCs and PCMT, trained on positively and negatively correlated patients achieved higher prediction accuracy than the classifiers trained on only positively correlated patients for all three cross-validation schemes.

**Figure 6:**
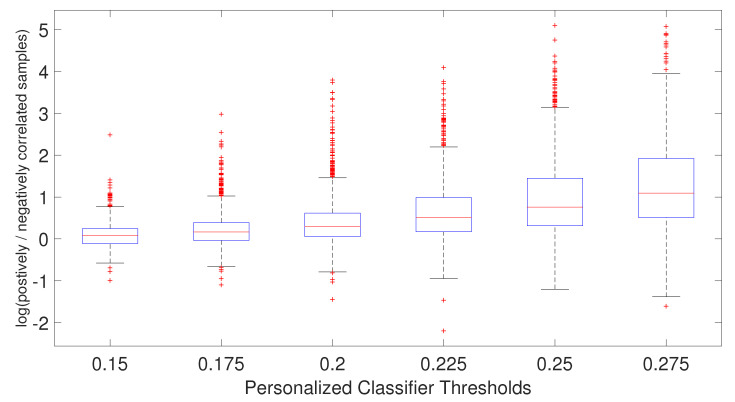
Boxplot of the ratio of positively and negatively correlated samples for each patient for each personalized classifier thresholds.

As shown in Figure 6, the ratio of positively and negatively correlated samples increases as the PCC threshold increases. In fact, when PCC = 0.275, not many negatively correlated samples can be found for the personalized classifiers. This explains why the AUC difference for PCC threshold 0.275 is very small between the personalized classifiers trained on positively and negatively correlated samples and personalized classifiers trained on only positively correlated samples. In other words, for PCC = 0.275, the personalized classifiers using positively and negatively correlated samples are almost identical to the personalized classifiers using only positively correlated samples, with very few negatively correlated samples remaining for that stringent PCC threshold. Taken together, it is evident that increasing patient diversity within the training data by including some negatively correlated samples has resulted in better prediction accuracy for the personalized classifiers.

### 3.4. Prediction Performance of Subtype Specific Classifiers

**Figure 7:**
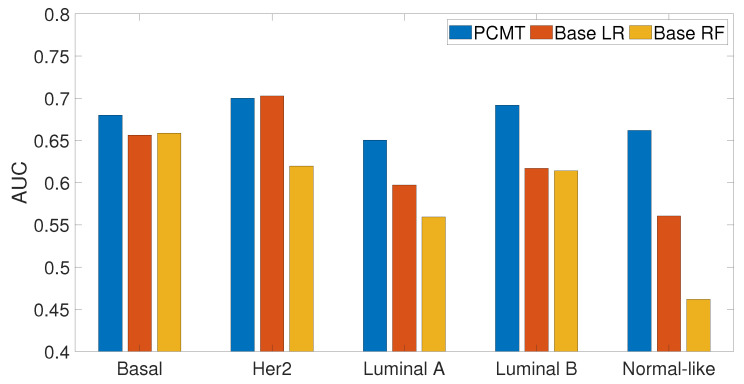
Prediction accuracy of subtype specific classifiers for PCMT, base LR and base RF models.

In this analysis, we examined how personalized classifiers compare to subtype-specific models built using patients belonging to a specific subtype. The prediction accuracy of PCMT, subtype-specific LR and subtype-specific RF classifiers are given in Figure 7. It can be observed that PCMT outperforms subtype-specific models in every subtype except HER2, where subtype-specific LR and PCMT achieved similar accuracy.

**Figure 8:**
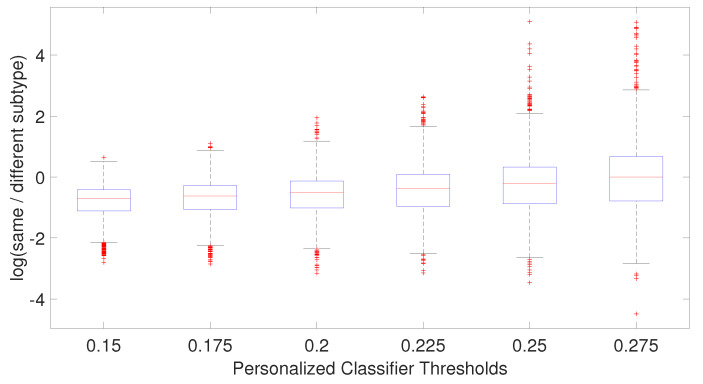
Boxplot of the ratio of patient subtype and other subtypes for each patient for each personalized classifier thresholds.

The relatively mediocre prediction accuracy of PCMT in HER2 is likely because the personalized classifiers included much more negatively correlated samples than positively correlated samples (data not shown), hampered the prediction accuracy of PCMT in the HER2 subtype. It is also observable that the patients in the HER2 subtype have lower correlations within themselves and have much higher negative correlations with the patients of different subtypes (Figure 4). In general, the training data for each personalized classifier included not only samples from the same subtype but also patients from different subtypes (Figure 8). For lower PCC thresholds, many patients have a similar ratio of the same/different subtypes in their classifiers, which helped improve prediction accuracy in these subtypes. It is important to note that subtype-specific LR and RF classifiers achieved poor prediction accuracy compared to PCMT in the normal-like subtype. This is likely because of two reasons. First, the number of patients in the normal-like subtype is smaller than in other subtypes. Secondly, the normal-like subtype is relatively less well-defined. PCMT achieved better prediction accuracy in the normal-like subtype with the help of including patients not strictly falling into the predefined normal-like subtype. It is worth noting that base LR outperforms base RF significantly in HER2, luminal A, and normal-like subtypes. Subtype-specific patients are much more similar to each other, so it has lower diversity within the dataset, potentially enabling base LR to obtain better prediction accuracy than base RF.

### 3.5. Robustness of Personalized Classifier

To evaluate whether the features selected by the classifiers are robust, we partitioned the dataset into two disjoint groups, applied different classification algorithms to each partition to select a given number of top features, and counted the number of common top features shared between the two partitions. Model robustness is then defined as the ratio between the observed number of common features and the expected number of common features (see Methods).

**Figure 9:**
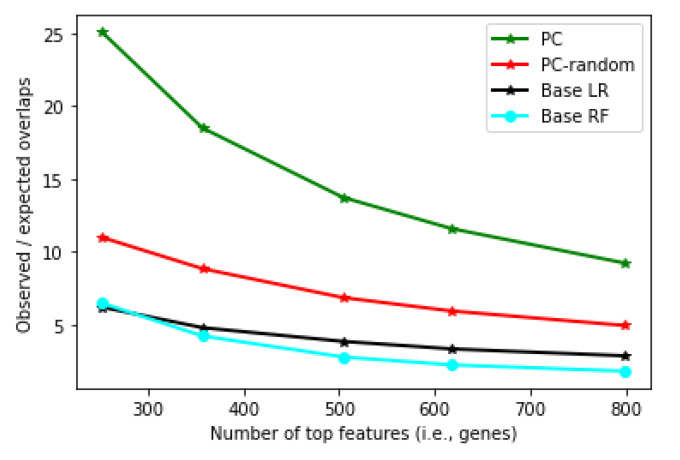
Robustness measure (y-axis) for Personalized LR, Base LR and Base RF at different numbers of top features (x-axis).

Figure 9 shows the robustness measure of the personalized classifier (PC) constructed with Pearson correlation threshold 0.175 (results with other thresholds are similar). It can be seen that the features obtained from PC are much more robust than that from the LR and RF classifiers. (Note that every classifier tested here has identified robust features, and the overlap is statistically significant in every classifier, with *p*-value $<10^{-17}$, Fisher’s exact test). Interestingly, when neighbors were selected randomly (PC-random, Figure 9), PC still showed better robustness than LR and RF, although at a much lower level compared to the real PC. This shows that the robustness achieved by PC is not simply because the feature scores from PC are based on an average of many classifiers (one for each patient).

**Figure 10:**
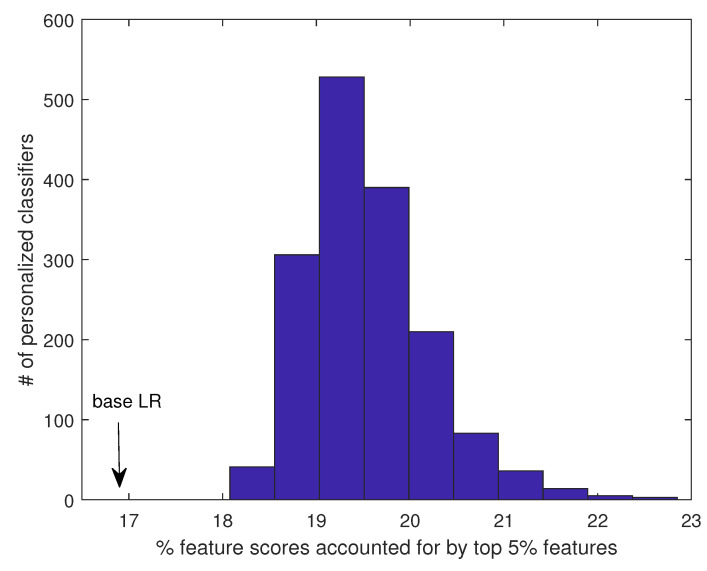
Percent of total feature scores accounted for by the top 5% features in PC vs. base LR.

The robustness of the PC can be attributed to the fact that each component classifier of the PC is learned from a group of generally very similar patients, and therefore, the feature scores are concentrated on relatively fewer features. As a result, the feature score distribution is skewed, with the important features scored much higher than the rest of the features, and feature ranking is robust between the two disjoint patient partitions. In contrast, when the classifier is learned from all patients (as in base LR), due to the high heterogeneity of the training samples, the decision boundary tends to be complex, and many more features are needed to construct a good model. Therefore, the feature scores tend to spread out to many features, which causes the feature ranking to be more sensitive to a small perturbation in the training data. To further explore this possibility, we computed the percent of total unsigned feature scores accounted for by the top 5% features. (Results on top 1% or 10% features are qualitatively similar.) For an LR model learned from all patients, the top 5% of features accounted for 16.9% of total feature weights. In comparison, for PC, the top 5% of features accounted for 19.6% of feature weights on average (minimum: 18.1%, maximum: 22.9%), significantly higher than in the base LR (Figure 10). Importantly, when the feature scores from the different patients were averaged, the same pattern remained: the top 5% of features accounted for 19.5% of feature weights. Together, these results support that the features identified by PC are likely true metastasis-related biomarkers for individual patients, which can be explored further to understand the molecular mechanism of metastasis and identify personalized druggable targets.

### 3.6. Top Genes Selected by Personalized Classifier

A key advantage of our algorithm is its ability to identify patient-specific features that are important for predicting individual metastasis events. For a more concise analysis, here we report the most significant features for each subtype by averaging the feature coefficient vectors belonging to patients in the same subtype. Table 2 shows the top 20 genes identified for each subtype by personalized classifiers and the top 20 features identified by LR from the whole dataset, while many genes have a clear association with cancer or metastasis, for a relatively fair comparison, we performed a PubMed query using the gene symbol, which is limited to title only, and the word “metastasis” or “metastatic”, which can appear in title or abstract, as keywords. The number of PubMed hits was recorded to indicate the extent of evidence for the gene’s association with metastasis.

From Table 2, it can be seen that overall, the personalized classifiers identified more known metastasis-associated genes than the base LR classifier (17–18 for personalized classifiers vs. 13 for LR). In addition, it appears that some of the top genes identified by LR may be associated with the metastasis of different subtypes. For example, ALB is a top feature in four other subtypes but not Basal. On the other hand, NTS, PTX3, and DLX2 are specific to the Basal subtype. In addition, the personalized classifiers identified many more markers that are not ranked high in LR. For example, CXCL8 is a well-known chemokine to modulate tumor proliferation, invasion, and migration [30], and has recently been identified as a biomarker for triple-negative (basal/basal-like) breast cancer [31,32]. Similar roles have also been identified for CXCL13 and UCHL1. Several well-known genes, such as MMP1, ALB, and AGR2, have been identified as among the top features for all subtypes except basal but were all missed by LR. CDH1, TFF1, and TFF3 have been found to be associated specifically with the HER2 subtype. GJA1, a key component in gap junction transmembrane channel, is a top feature in Luminal A, Luminal B, and normal-like subtype, consistent with a recent finding [33].

### 3.7. Potential Challenges and Limitations for Applications to Other Cancer Types

Since most types of cancer are heterogeneous in nature, and have multiple subtypes that have different molecular and/or clinical characteristics [34], we believe the general idea proposed in this paper may have the potential to be applied to other cancer types for predicting metastasis or other clinical endpoints. However, some of the design principles in our method implicitly utilize key insight from breast cancer subtypes, for example, the approximate subtype distributions, which can vary between different cancer types. Therefore, some fine tuning of the parameters may be necessary and may depend on cancer-specific characteristics. In addition, to learn personalized models requires a relatively larger number of training samples to perform sample selection. We found that newer dataset, such as the expression data collected by The Cancer Genome Atlas [26], do not yet have enough labelled patients to train personalized models, as the cancer outcomes for many patients are censored. Therefore, we plan to evaluate the effectiveness of the method on other cancer types in a future study.

## 4. Conclusions

Improving the prediction of metastasis is crucial to reduce the death risk of breast cancer patients. In this paper, we proposed training personalized classifiers using only a subset of training samples whose gene expression levels are highly correlated with the test patient. Results show that the personalized classifier obtained better prediction accuracy than the base models trained on the complete training dataset as well as models trained with patient in the same intrinsic subtype. Our results also revealed that including negatively correlated patients in the personalized classifier improves the diversity within the model, leading to more accurate predictions when compared to classifiers trained on only positively correlated patients. The top features identified by the personalized models were shown to be more robust than those identified by the base models. Moreover, the personalized models identified different top features for different subtypes, some of which were already shown to be associated with subtype-specific metastasis. It is worth noting that these results are based on one of the largest datasets for breast cancer metastasis prediction consisting of 12 patient cohorts, as well as two additional datasets that have been processed separately. In particular, the cross-dataset evaluation based on TCGA breast cancer data is challenging, as the models were trained on microarray data and tested on RNAseq data. Therefore, the results support that our method has achieved genuine, robust performance gain compared to traditional methods in predicting breast cancer metastasis. We believe that, with sufficient training data and further parameter tuning, the idea of personalized classifiers may be generalized to other applications, such as predicting clinical endpoints for other cancer types and designing personalized medicine for cancer patients.

## Figures and Tables

**Table 1:** Specification of the studies in ACES.

Dataset	Geo Accession No.	No. of Poor	No. of Good	Total Patient
Desmedt	7390	56	127	183
Hatzis	25,066	102	48	150
Ivshina	4922	30	72	102
Loi	6532	24	33	57
Pawitan	1456	33	114	147
Miller	3494	21	68	89
Minn	2603	21	44	65
Schmidt	11,121	24	145	169
Symmans	17,705	37	187	224
WangY	5327	10	42	52
WangYE	2034	88	169	257
Zhang	12,093	9	112	121
ACES		455	1161	1616

**Table 2:** Top 20 genes from each breast cancer subtype are given in the table for personalized classifier from LOOCV (PCC threshold = 0.2). The top 20 genes from Base LR are also given. Hits indicate that with that gene symbol, the number of PubMed hits occurred with the keyword metastasis/metastatic in the title/abstract of articles within the PubMed database. Genes are sorted by PubMed hits.

Top Genes from Personalized Classifier for Each Subtype										Top Genes from LR	
Basal		HER2		Luminal A		Luminal B		Normal-like			
**Gene**	**Hits**	**Gene**	**Hits**	**Gene**	**Hits**	**Gene**	**Hits**	**Gene**	**Hits**	**Gene**	**Hits**
CXCL8	55	MMP1	280	AGR2	44	MMP1	280	MMP1	280	KIT	393
CXCL13	25	CDH1	172	ALB	39	AGR2	44	CD24	236	ALB	39
UCHL1	22	AGR2	44	OLFM4	14	ALB	39	ESR1	138	NTS	22
PTX3	13	ALB	39	GJA1	10	TFF1	25	AGR2	44	PTX3	13
NTRK2	7	TFF1	25	HOXC10	5	GJA1	10	ALB	39	DLX2	3
SEMA3C	6	TFF3	20	TSPYL5	3	DUSP4	4	OLFM4	14	PBX3	3
DUSP4	4	OLFM4	14	HLA-DQB1	3	CST1	4	STC1	13	HMGCS2	2
CST1	4	AZGP1	8	TMPRSS3	2	TSPYL5	3	GJA1	10	COL4A6	2
TSPYL5	3	CST1	4	HOPX	2	HLA-DQB1	3	KRT7	7	HOXB2	1
DLX2	3	TSPYL5	3	CPB1	1	HMGCS2	2	CLCA2	3	SPON1	1
ZIC1	2	HLA-DQB1	3	NPY1R	1	CPB1	1	HMGCS2	2	TMEM47	1
TMPRSS3	2	HMGCS2	2	SLC1A1	1	NPY1R	1	CPB1	1	ANK3	1
CPB1	1	CPB1	1	DDIT4	1	TFAP2B	1	SCGB2A1	1	MYO6	1
NPY1R	1	DDIT4	1	SCGB2A1	1	DHRS2	1	UGT2B4	1	ZDHHC11	0
SLC1A1	1	TFAP2B	1	GRIA2	1	DDIT4	1	DDIT4	1	CRISP2	0
TMEM47	1	SCGB1D2	1	SCGB1D2	1	SCGB1D2	1	TFAP2B	1	QDPR	0
GPRC5B	1	DHRS2	1	MSMB	1	SLC1A1	1	GRIA2	1	MNDA	0
MYBPC1	0	VGLL1	1	TFAP2B	1	CSN3	0	PDZK1	1	GSTT2	0
CHPT1	0	CYP4B1	0	TCN1	0	KRT15	0	TCN1	0	MREG	0
FMO3	0	TCN1	0	MYBPC1	0	PGGHG	0	UGT2B28	0	C3orf14	0

## Data Availability

Software code and associated data sets are available upon request from the corresponding author.
